# Soil Water Availability Modulation Over Estimated Relative Yield Losses in Wheat (*Triticum aestivum* L.) Due to Ozone Exposure

**DOI:** 10.1100/tsw.2007.120

**Published:** 2007-05-29

**Authors:** Daniel De la Torre, Maria Jose Sierra

**Affiliations:** ^1^ Ecology Department, Pharmacy Building, Campus Miguel de Unamuno, University of Salamanca, 37008 Salamanca, Spain; ^2^ Environment Department, Unit of Evaluation and Control of Conventional Contamination, CIEMAT, Avenida Complutense 22, 28040 Madrid, Spain

**Keywords:** Mediterranean conditions, ozone, soil water availability, wheat, yield losses

## Abstract

The approach developed by Fuhrer in 1995 to estimate wheat yield losses induced by ozone and modulated by the soil water content (SWC) was applied to the data on Catalonian wheat yields. The aim of our work was to apply this approach and adjust it to Mediterranean environmental conditions by means of the necessary corrections. The main objective pursued was to prove the importance of soil water availability in the estimation of relative wheat yield losses as a factor that modifies the effects of tropospheric ozone on wheat, and to develop the algorithms required for the estimation of relative yield losses, adapted to the Mediterranean environmental conditions. The results show that this is an easy way to estimate relative yield losses just using meteorological data, without using ozone fluxes, which are much more difficult to calculate. Soil water availability is very important as a modulating factor of the effects of ozone on wheat; when soil water availability decreases, almost twice the amount of accumulated exposure to ozone is required to induce the same percentage of yield loss as in years when soil water availability is high.

### INTRODUCTION

Tropospheric ozone has been shown to induce important yield losses in cereal crops[1,2,3]. One negative aspect of the exposure of cereal crops to ozone is yield loss. In wheat, this can be seen as decreasing spike numbers per surface unit, decreasing grain numbers per spike, and a decrease in grain weight, with the concomitant decrease in spike weight and size[4,5,6,7,8].

In the past few decades, the impact of tropospheric ozone exposure on plants has been one of the most important environmental issues considered by the European cooperation panel on air pollution emissions control[9]. This has led to a request from policymakers to scientists for methods to quantify these effects.

Earlier experiments with ozone and wheat (*Triticum aestivum* L.) carried out under semi-natural conditions in open-top chambers (OTCs) and involving several wheat varieties in different countries, such as the U.S.[10], Switzerland[1,11], Finland[12], Denmark[13], and Sweden[14,15], have reported very consistent responses to ozone by crops, in such a way that these experiments have become the basis for the determination of the critical ozone level by means of the accumulated ozone threshold above 40 nl $l^{-1}\cdot h$ (AOT40) over a period of 3 months (from anthesis to harvest), which would produce a 5% relative yield loss. From the regression equation that relates wheat yield to ozone exposure, the AOT40 value predicted was 3000 nl $l^{-1}\cdot h$, and this has been considered as the critical ozone level for crop protection[16].

The use of OTCs may give rise to certain problems, such as a higher ozone absorption by plants due to higher relative humidity and no watering limitations inside the chamber; soil water limitations lead to lower ozone absorption because stomatal conductance declines[17,18]. Additionally, the sensitivity to ozone of the varieties used in these experiments could vary with respect to those used regionally by farmers. All these empirical limitations mean that direct estimation of the relative economic losses of wheat crops using the model proposed by Fuhrer et al. in 1997[16] would probably afford an overestimation of the effects of ozone expressed as relative yield losses, especially in Mediterranean countries, where cereal crops are mostly not irrigated and rainfall is usually much lower than in Northern and Central Europe, where crops are sometimes irrigated during drought periods[19].

Indeed, a problem of overestimation arises when this model is applied for the determination of critical ozone levels when soil water availability is a limiting factor[16,19,20]. It has been stated explicitly that this function cannot be used directly for the estimation of relative economic losses[21,22,23].

In order to avoid this problem of overestimation, two different approaches have been proposed. The first — currently adopted by the EMEP-CLRTAP (European Monitoring and Evaluation Programme – Convention on Long Range Transboundary Air Pollution) — consists in determining plant ozone absorption and set dose-response functions instead of exposure-response functions. The second consists in correcting the deviations in relative yield loss calculated with dose-response functions by introducing certain environmental factors that modify crop ozone uptake in the calculations; thus, the correction would imply the building of empirical relationships for each environmental factor and the selection of those that can be applied to the dose-response function proposed by Fuhrer in 1995[17].

Dose-response functions can be considered a better indicator than those of exposure-response in the assessment of the risk of plant damage[20,24] since the former are more realistic in that they consider how environmental factors affect plant ozone uptake. Nevertheless, calculation of the ozone dose is based on stomatal fluxes, whose determination requires the use of micrometeorological observations, in most cases unavailable. It may be of interest, therefore, to develop alternative approaches, such as the one proposed by Fuhrer in 1995[17].

Environmental factors may affect plant ozone uptake; in particular, vapor pressure deficit (VPD) and soil water content (SWC) play a crucial role in the sense that they affect plant stomatal conductance and, hence, the gaseous exchange between the atmosphere and the vegetation, especially in southern Europe where dryer conditions tend to prevail[25]. In 1995, Fuhrer[17] proposed a methodology that evaluates the impact of ozone on wheat crops by means of the modification exerted by the SWC on wheat yield loss due to ozone, using an earlier empirical dose-response model[11]. That author developed a methodology to calculate a modifying factor that uses the modulation exerted by soil water availability, or soil water content (SWC), on the impact of ozone on wheat yield, called $f_{water}$ ($f_w$); this factor was calculated for the 2 months preceding harvest (May–June), i.e., from anthesis to harvest, since this period has been considered to be the most sensitive to ozone because of the repercussions of this gas on wheat yield[26,27,28].

In this study, we want to prove the effectiveness of Fuhrer's approach for Mediterranean wheat, making the necessary adjustments to the algorithms for the calculation of relative yield loss.

### MATERIALS AND METHODS

The model proposed by Fuhrer in 1995[17] calculates $f_w$ from the data on potential evapotranspiration (PET) and rainfall. It also uses a database with wheat yield data and environmental data, such as air temperature, solar radiation, and rainfall (see Appendix, Tables 1–7).

#### Wheat Yield Database

A yield (kg/ha) database was built from four commercial winter wheat cultivars (cv) (*Triticum aestivum* L.) — namely, “Etecho”, “Marius”, “Soissons”, and “Tremie” — for six experimental campaigns (1994–2000) and six experimental sites belonging to the experimental network (Xarxa d’experimentació varietal de cereals a Catalunya) managed by the IRTA (Institut de Recerca i Tecnología Agroalimentàries) with a view to evaluating the behavior of these varieties regarding ozone.

The six experimental sites belong to three Catalonian agroclimatic regions (see Fig. 1)[29] that differ in their mean annual rainfall and the height above sea level (h.a.s.l): Secans Semifrescals (500–600 mm, 500–700 h.a.s.l), Girona Interior (>700 mm, 50–500 h.a.s.l), and Secans Frescals (700–1000 mm, 500–700 h.a.s.l).

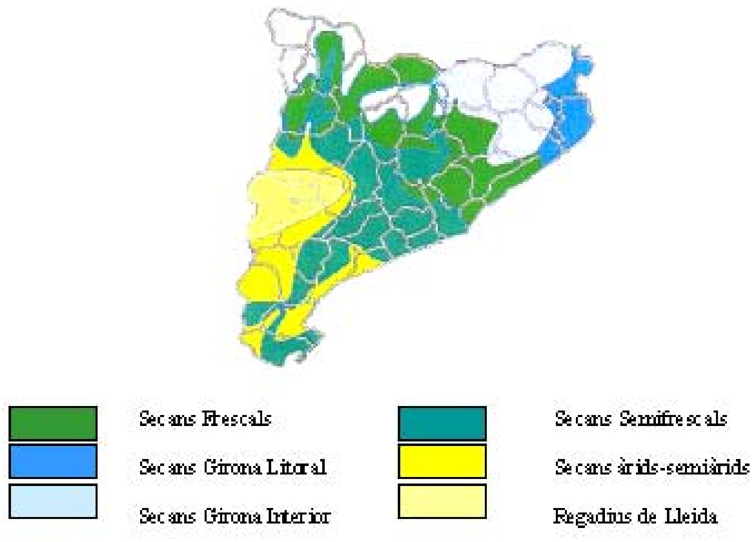

**FIGURE 1.** Catalonian agroclimatic regions. Another three regions are shown that were not included in the study owing to a lack of empirical data.

The yield data range was 2722–6200 kg/ha for Secans Semifrescals, 3458–8712 kg/ha for Secans Frescals, and 4406–8495 kg/ha for Girona Interior, and the relative yield range was 0.44–0.99 for Secans Semifrescals, 0.38–1.0 for Secans Frescals, and 0.49–0.95 for Girona Interior.

Three phenological periods were defined: from sowing to harvest (S–H, November–June), from sowing to anthesis (S–A, November–May), and from anthesis to harvest (A–H, May–June). The first two periods were selected because decreasing water availability during stem elongation, and during the corresponding phenological periods between flag leaf development and anthesis, may lead to appreciable reductions in wheat yield, seen as a decreasing amount of seeds[30]. The third period was considered due to the special sensitivity of wheat to ozone in the period from anthesis to harvest[26,28]. It is important to note that the A–H period is longer in Northern and Central Europe (May–July) than in Mediterranean countries (May–June), with a warmer and drier climate.

### Database of Meteorological Parameters.

Temperature (T), precipitation (N), and global radiation (R) data were used to calculate PET in the study zones; such data were recorded at the respective closest Catalonian Meteorological Network stations:

- Secans Semifrescals*:* Calaf, La Foradada, and Santa Coloma
- Secans Frescals*:* Solsona and Vic
- Girona Interior*:* Ruidellots de la Selva

The mean temperature ranges were 7–11ºC for the S–A period, 9–12ºC for the S–H period, and 15–21ºC for the A–H period. The accumulated global radiation range was 1990–2732 MJ $m^{-2}$ for S–A, 2535–3447 MJ $m^{-2}$ for S–H, and 1089–1514 MJ $m^{-2}$ for A–H. The accumulated precipitation range was 48–678 mm for S–A, 155–739 mm for S–H, and 0.2–168 for A–H.

### Calculation of PET

PET (mm/month) was determined with an empirical equation in which mean air temperature (T, ºC) and monthly global radiation (R, J $s^{-1}m^{-2}$) are needed as input data[17,31]:

$$PET = a\,(T + b)\,R\;8.64\;10^4\,s\;day^{-1}\;10^{-6}\,m^3\,g^{-1}/2430\;J\,g^{-1} \quad \text{(Eq. 1)}$$

where a = 0.025ºC$^{-1}$ and b = 3ºC.

The accumulated PET range was 282–407 mm for S–A, 412–581 for S–H, and 229–353 for A–H.

### Calculation of SWC (%FC).

SWC was calculated from the PET and precipitation (N) data for each month included in the study period[17], following Eq. 2:

$$\%FC = ([FC_o - B\,\{\sum PET - \sum N\}^{-1/2}]/FC_s)\;100 \quad \text{(Eq. 2)}$$

where B is equal to 1 when there is a vegetation canopy (in this case the wheat crop), $FC_o$ is the SWC at the beginning of the study period, and $FC_s$ is the SWC at field capacity. These values have been established at 28 and 35%, respectively, for soils destined for wheat cultivation[17].

The %FC range was:

- For the Secans Frescals region: 36–74% for S–A, 38–64% for S–H, and 36–51% for A–H
- For the Girona Interior region: 26–63% for S–A, 25–64% for S–H, and 36–52% for A–H
- For the Secans Semifrescals region: 38–66% for S–A, 27–75% for S–H, and 31–49% for A–H

### Calculation of the Correction Factor ($f_{water}$) based on the SWC

It was assumed that the effect of a decrease in SWC (%FC) on the sensitivity of plants to ozone uptake, expressed as $f_{water}$ ($f_w$), would be to reduce such uptake. The same effect is seen for the relative wheat yield (Y, %)[17], and hence one has $f_{water}$ = Y. This factor is equal to 1 when %FC > 60%, while for %FC < 60% $f_w$ would be calculated by means of the following empirical equation[17]:

$$f_w = Y = -0.455 + 0.024\,(\%FC)\;(R^2 = 0.89,\; n = 7) \quad \text{(Eq. 3)}$$

As well as calculating $f_w$ according to Eq. 3, this factor was also calculated from the Catalonian database of Y and %FC, then applying the several empirical regression equations found (see below).

### Calculation of Relative Wheat Yield (Y, %) Loss Induced by Ozone Exposure and Modulated by Soil Water Availability (%FC)

The effect of ozone on wheat yield was calculated using the equation developed by Fuhrer in 1997[16], where AOT40 values are expressed in nl $l^{-1}\cdot h$:

$$Y (\%) = 99.5 - 1.7\ AOT40;\ R^2 = 0.89\ (Eq.\ 4).$$

The relative yield loss would thus be equal to 100% – Y (%), although if SWC modulation is taken into account, this expression must be multiplied by the value of the $f_w$ correction factor:

$$Loss\ Y (\%) = (100 - Y)\ f_{water}\ (Eq.\ 5)$$

### Calculation of the Correction Factor ($f_{water}$) Based on SWC for Catalonian Wheat Yield Data

The empirical relationship proposed by Fuhrer in 1995[17] (see Eq. 3) was contrasted with the empirical relationships obtained with the wheat yield data from Catalonia.

A preliminary analysis of variance (ANOVA, StatSoft, Inc., 1996) was performed to determine whether there were significant differences in the wheat yield due to the characteristics of the agroclimatic region or those of the wheat cultivar. No significant differences were found in wheat yield due to the cultivar factor ($p = 0.28$, n = 108, F = 1.3). However, the existence of a significant gradient in wheat yield due to the agroclimatic factor was observed ($p < 0.01$, n = 108, F = 33.8). This was associated with the SWC, with higher yields in the more humid regions (Secans Frescals and Girona Interior). Thus, three empirical models were generated relating relative yield (Y, %) and SWC (%FC): the first for all three agroclimatic regions; the second for the more humid regions (Secans Frescals and Girona Interior), with no significant differences between them; and the third for the driest (Secans Semifrescals). Yield data were normalized with respect to the highest value recorded for each group.

Thus, with the three agroclimatic region groups and three periods considered, nine empirical models were built for the calculation of relative wheat yield (Y, %), expressed as the correction factor ($f_w$), as a function of the SWC (%FC).

These empirical models were expressed as simple linear regressions (Y = $f_w$ *vs*. %FC) whose determination coefficients ($R^2$) determined the periods where the correlation between wheat yield and SWC was maximum. Then, employing the empirical regressions obtained, the yield (Y) or $f_w$ variation range was estimated for each agroclimatic region group and each period considered as a function of the minimum and maximum SWC (%FC) values.

## RESULTS

### Empirical Models Relating Relative Yield (Y, %) to SWC (%FC)

The results show that the Catalonian agroclimatic regions considered are suitable for the objectives of the study, since a wheat yield gradient was observed as a function of SWC. The least humid region, Secans Semifrescals, showed the lowest yield.

Table 1 shows the results of the regression analysis for relative yield (Y, %) *vs*. SWC (%FC) performed in order to determine when wheat plants are most sensitive to soil water deficits.

**TABLE 1**
**Results of Regression Analysis for Relative Wheat Yield (Y) and SWC (%FC)**

| | S–H | A–H | S–A |
|---|---|---|---|
| All regions | $R^2$ = 0.44, n = 91, $p$ < 0.05 | $R^2$ = 0.36, n = 88, $p$ < 0.05 | $R^2$ = 2.1e-3, n = 97, n.s. |
| Secans Frescals + Girona Interior | $R^2$ = 0.24, n = 57, $p$ < 0.05 | $R^2$ = 0.17, n = 56, n.s. | $R^2$ = 0.11, n = 64, n.s. |
| Secans Semifrescals | $R^2$ = 0.25, n = 26, $p$ < 0.05 | $R^2$ = 0.41, n = 32, $p$ < 0.05 | $R^2$ = 0.69, n = 33, $p$ < 0.05 |

### Calculation of $f_{water}$

The correction factors ($f_w$) were calculated using Fuhrer's equation[17](see Eq. 3) and using the regression relations obtained for the empirical data for Catalonia; i.e., for all the regions together, for the more humid regions (Secans Frescals + Girona Interior) and for the least humid region (Secans Semifrescals)(see Eqs. 6–9; Table 2). Fuhrer's approach[17] was designed for the A–H period and, hence, this same period was considered for the empirical relations found. Additionally, the S–A period was considered for the least humid region, Secans Semifrescals, because this period was the one that best explained the wheat yield for this region (see above).

**TABLE 2**
**Equations for the Calculation of $f_{water}$**

| Region | Period | %FC Coefficient | Independent Term | $R^2$ | *p* Value |
|---|---|---|---|---|---|
| Fuhrer's model (Eq. 3) | A–H | 0.024 | –0.455 | 0.89 | <0.05 |
| All regions (Eq. 6) | A–H | 0.0136 | 0.102 | 0.36 | <0.05 |
| Secans Frescals + Girona Interior (Eq. 7) | A–H | 0.0126 | 0.1433 | 0.17 | n.s. |
| Secans Semifrescals (Eq. 8) | A–H | 0.0181 | –0.0618 | 0.41 | <0.05 |
| Secans Semifrescals (Eq. 9) | S–A | 0.0142 | –0.0643 | 0.69 | <0.05 |

**TABLE 3**
**$f_{water}$ Variation Range Considering the Maximum and Minimum %FC Values for Each Group of Agroclimatic Regions and the Corresponding Phenological Periods**

| | Fuhrer's $f_w$ (A–H) | Empirical Relationship for All Regions (A–H) | Empirical Relationship for the More Humid Regions (A–H) | Empirical Relationship for the Least Humid Region (A–H) | Empirical Relationship for the Least Humid Region (S–A) |
|---|---|---|---|---|---|
| $f_{water}$ range | 0.16–1 | 0.52–0.73 | 0.48–0.88 | 0.47–0.82 | 0.58–0.74 |

*Note:* $f_{water}$ value range obtained with Fuhrer's equation[17].

Table 3 shows the $f_w$ (Y = $f_w$) variation range calculated according to Eqs. 3 and 6–9 (see Table 2), taking the corresponding maximum and minimum SWC (%FC) values for each group of agroclimatic regions and the above-specified phenological periods.

Contrary to what occurs in Switzerland, in Catalonia, the SWC (%FC) is seldom higher than 60% and so relative wheat yield would seldom reach maximum values.

### Calculation of Relative Wheat Yield Loss Corrected by $f_{water}$

The $f_w$ values obtained were employed to modulate the estimation of the effect of ozone exposure on wheat yield using Eqs. 4 and 5, where ozone exposure is expressed as the AOT40 index (nl $l^{-1}$·h) accumulated over 3 months (see Table 4).

**TABLE 4**
**Estimation of Relative Wheat Yield Losses (%) Corresponding to Several Ozone Exposures and to the $f_w$ value Range Shown in Table 2**

| AOT40 (nl $l^{-1}$·h) | Exposure-Response[a] (Eq. 4) | $f_w$ Correction (A–H)[b] (Eq. 5) | More Humid Region Model (A–H)[c] (Eq.7 ) | Least Humid Region Model (A–H)[d] (Eq. 8) | Least Humid Region Model (S–A)[e] (Eq. 9) |
|---|---|---|---|---|---|
| 3000 | 5.6 | 0.9–5.6 | 2.9–4.1 | 2.6–4.6 | 3.2–4.1 |
| 6000 | 10.7 | 1.7–10.7 | 5.6–7.8 | 5.0–8.8 | 6.2–7.9 |
| 9000 | 15.8 | 2.5–15.8 | 8.2–11.5 | 7.4–13.0 | 9.2–11.7 |
| 12,000 | 20.9 | 3.3–20.9 | 10.9–15.3 | 9.8–15.3 | 12.1–15.5 |

*Note:* Relative yield losses following: [a]the exposure-response function[11], [b]Fuhrer's correction[17] (A–H), [c]the more humid regions model (A–H), [d]the least humid region model (A–H), [e]the least humid region model (S–A).

**TABLE 5**
**AOT40 (nl $l^{-1}$·h) over 3 Months that would Induce Relative Yield Losses of 5, 10, and 20%, Respectively**

| Loss Y (%) | AOT40 (nl $l^{-1}$·h)* | AOT40 (nl $l^{-1}$·h); More Humid Regions (A–H) | AOT40 (nl $l^{-1}$·h); Least Humid Region (A–H) | AOT40 (nl $l^{-1}$·h); Least Humid Region (S–A) |
|---|---|---|---|---|
| 5 | 2647 | 5833–3048 | 5964–3293 | 4777–3680 |
| 10 | 5320 | 11,960–6879 | 12,222–6879 | 9848–7655 |
| 20 | 11,470 | 24,216–13,075 | 24,737–14,053 | 19,990–15,604 |

* Fuhrer's equation[16] with no correction for the SWC.

### Estimation of Accumulated Exposure to Ozone (AOT40) Required Inducing Specific Relative Yield Losses

The AOT40 values associated with a given relative wheat yield loss may vary strongly, depending on soil water availability. Table 5 shows the AOT40 values required to induce relative yield losses of 5–20%. Calculations were accomplished using Eq. 4[16] and by combining Eq. 4 with the equations derived from the empirical data for the regions and periods considered (see Eqs. 6–9, Table 2). Equation 5 was used to estimate the relative yield from specific yield losses, and from the SWC correction factor ($f_w$) thus:

$$Y (\%) = 100 - (\text{LossY})/f_w \text{ (Eq. 5)}$$

where $f_w$ is taken from Table 3 and Y is used in Eq. 4 to estimate the AOT40 value needed to induce a specific relative wheat yield loss:

$$\text{AOT40 (nl } l^{-1}\cdot\text{h)} = (99.5 - Y)/1.7 \text{ (Eq. 4)}$$

## DISCUSSION

### Empirical Models Relating Relative Yield (Y, %) to SWC (%FC)

When the data for all three regions were considered together, the SWC accounted for yield to almost the same extent for both the S–H and the A–H periods ($R^2$ = 0.44, $p < 0.05$; $R^2$ = 0.36, $p < 0.05$). Nevertheless, no significant relationship was observed on considering the S–A period. It would appear that soil water availability in the A–H period is determinant for predicting the relative wheat yield. The same pattern was obtained on considering the more humid regions (Secans Frescals + Girona Interior) for the same periods, respectively, although the explanation of the variance was not very good ($R^2$ = 0.24, $p$ <0.05; $R^2$ = 0.17, n.s.; respectively), probably because the soil humidity gradient was very weak. Accordingly, the regression is not significant for the A–H period.

When the data for the least humid region (Secans Semifrescals) were analyzed, an inverse pattern was observed; i.e., a good correlation for the A–H period ($R^2$ = 0.41, $p < 0.05$), although the best correlation was found for the S–A period ($R^2$ = 0.69, $p < 0.05$), indicating that the wheat yield in this region is very sensitive to soil water availability during the vegetative period (from sowing to anthesis). This is in agreement with the results obtained by different authors over a broad range of environmental conditions[30,32,33,34,35]. This result is very interesting because the agroclimatic region addressed here represents the Mediterranean environmental conditions that predominate for most wheat crops in Central and Southern Spain, which favor early crop maturation. The approach developed by Fuhrer in 1995[17] was used with wheat yield data from Switzerland, where the environmental conditions are similar to those prevalent in the more humid regions considered in this study. By contrast to the S–A period, during the A–H period, temperature and solar radiation often increase, while rainfall and soil water availability decrease, favoring stomatal closure and hence a decrease in ozone uptake[17,18].

The carbohydrates stored in the periods before anthesis are extremely important for final grain yields in Mediterranean conditions[8,36], and hence the importance of the S–A period.

Thus, a global analysis of the results shows that Fuhrer's approach[17] should be modified for application in Mediterranean environmental conditions because wheat yield is very sensitive to soil water availability during the vegetative period (S–A), in contrast with its maximum sensitivity to ozone during the reproductive and maturation periods (A–H). This modification would be achieved by means of the new empirical models relating relative yield (Y, %) to SWC (%FC), found for the Catalonian data.

### Calculation of Relative Wheat Yield Loss Corrected by $f_{water}$

On comparing Fuhrer's approach[17] with the empirical model found for the more humid regions (Secans Frescals + Girona Interior) in the A–H period, it was observed that Fuhrer's model estimates lower relative yield losses (2–7%) induced by ozone for the drier years (minimum %FC and $f_w$ values) and that it estimates higher relative yield losses for the more humid years (1.5–5.6%) relative yield losses. Similar results were obtained on comparing Fuhrer's approach with the empirical models for the least humid region (Secans Semifrescals) for both the A–H and A–S periods.

The greatest relative yield losses correspond to the least humid region, Secans Semifrescals, for the S–A period (the period that best fits for this region), increasing with the rise in ozone exposure, with the highest yield loss value for 12,000 nl $l^{-1}\cdot$h of AOT40 accumulated exposure.

As seen in Table 4, the relative yield losses for this region are very similar for both periods, A–H and S–A, mainly in years with high soil water availability. Additionally, the relative yield losses estimated for the more humid regions with high soil water availability in the A–H period are almost the same as those predicted for the least humid region for the S–A period, with low soil water availability. In other words, it seems that no significant differences in relative yield losses between the two regions would be found. Nevertheless, the most significant model was that developed for the least humid region (Secans Semifrescals) in the S–A period.

For Mediterranean environmental conditions, the AOT40 value for 2 months is about 3000 nl $l^{-1}$·h, which (as seen in Table 4) would mean about 3–4% of relative yield losses, while according to Fuhrer[16] (see Eq. 4) they would be about 6%. In comparison with the models developed empirically (see Table 4), Fuhrer's model[17] — taking into account the SWC correction factor — underestimates for the least humid conditions (about 1% of relative yield loss) and overestimates for the most humid conditions (about 5.5% of relative yield losses). This means that Fuhrer's model[17] must be adapted for Mediterranean environmental conditions.

Relative wheat yield losses of around 6–12% (see Table 4) are expected for an AOT40 of 6000–9000 nl $l^{-1}$·h for the least humid region, in agreement with recent results by several authors[37], who estimated relative yield losses for wheat of 5–15% for those mean annual AOT40 values.

### Estimation of AOT40 Required Inducing Specific Relative Yield Losses

Table 5 shows that when soil water availability is taken into consideration as a correction factor, the AOT40 value varies considerably for the same percentage of relative yield losses, greater ozone exposure being required to induce the same relative yield loss for minimum SWC (minimum $f_w$) than that required for maximum SWC (maximum $f_w$).

Since the AOT40 values needed to induce a given relative yield loss for each region and period are higher when soil water availability is lower (lower $f_w$) — indeed, for dry years they are almost double than those for humid years — it could be suggested that when soil water availability is lower, higher ozone exposure (almost double) would be required to induce the same relative yield loss as under conditions without soil water deficit, in agreement with some authors[17,18]. This is why the current critical level of 3000 nl $l^{-1}$·h, which would induce relative yield losses of 5%, may be excessively strict for wheat crops grown in typical Mediterranean environmental conditions, where situations of soil water deficit tend to be the norm.

Although yield relationships as a function of SWC differ depending on the agroclimatic region, the ozone exposure needed to induce a specific relative yield loss is similar for both groups of regions when considering the same period (A–H). This is in agreement with the results shown in Table 4, where similar relative yield losses are expected for specific AOT40 values for both groups of regions (the most humid, Secans Frescals and Girona Interior, and the least humid, Secans Semifrecals) in the A–H period. Nevertheless, on considering the least humid region in the S–A period, when the Y *vs.* %FC regression relationship is best fitted, the AOT40 to induce a specific relative yield loss is lower (almost half the amount) than the AOT40 required to induce the same loss in the most humid regions for the A–H period. This is probably because it is during the reproductive period (A–H) when stomatal conductance and ozone uptake are highest[26,28]. Again, the importance of adjusting the phenological period for the estimation of the relative wheat yield losses, depending on the environmental conditions, can be appreciated.

Another important adjustment is the period considered for accumulated ozone exposure and the establishment of the critical ozone level. The period used here was from May to July, corresponding to the typical reproductive phenological period for wheat in Northern and Eastern Europe, although the precociousness of Mediterranean wheat is higher, maturation usually ending in June. Thus, the 2-month period used to calculate the AOT40 critical level should extend from May to June in Mediterranean environmental conditions.

### CONCLUSIONS

The methodology proposed by Fuhrer has great potential for a realistic estimation of the relative wheat yield losses induced by ozone exposure; it involves the modulation exerted by soil water availability in the uptake of this pollutant.

Fuhrer's approach can be applied to Mediterranean environmental conditions, but in order to obtain more precise and accurate relationships between relative yield (Y, %) and the SWC (%FC), new empirical relationships should be obtained owing to the underestimation in Fuhrer's method of the relative yield losses for low SWCs (2–7% less) and the overestimation for high SWCs (2–6% more).

The phenological periods considered (vegetative and reproductive periods) should be adjusted for the prevailing environmental conditions when performing the algorithms for the estimation of the correction factor ($f_w$).

Soil water availability, expressed as SWC (%FC), is very important as a modulating factor of the effects of ozone on wheat; when soil water availability decreases, almost twice the amount of accumulated exposure to ozone, expressed as AOT40 (nl $l^{-1}$·h), is required to induce the same percentage of yield loss as in years when soil water availability is high.

Finally, it could be suggested that in Mediterranean environmental conditions, where ozone concentrations tend to be high (especially in spring and summer), due to the inverse modulation exerted by the SWC, the true relative yield losses would be lower than in other environmental conditions where soil water availability is higher.

### ACKNOWLEDGMENTS

Acknowledgment is given to *TheScientificWorldJOURNAL* Editor responsible for handling this manuscript. Thanks are given to Dr. Joan Serra, researcher at IRTA (Institut de Recerca i Tecnología Agroalimentàries), who kindly provided the wheat yield data of Catalonia for 6 years (1994–2000). We are also very thankful to D. Sergi Paricio i Ferreró, Director of the Catalonian Meteorological Service, belonging to the "Dirección General de Qualitat Ambiental, Departament de Medi Ambient", who provided the meteorological data for the experimental campaigns.

## APPENDIX

**TABLE 1**
**Yield, Relative Yield, and SWC (%FC) Data for Every Agroclimatic Station, Wheat Variety, and Year for the Agroclimatic Region Secans Frescals**

| Station | Variety | Year | Yield (kg/ha) | Y | %FC (S–A) | %FC (S–H) | %FC (A–H) |
|---|---|---|---|---|---|---|---|
| Solsona | ETECHO | 95–96 | 7538 | 0.84 | 36.15 | 42.2172 | 51.52 |
| | | 96–97 | 7545 | 0.84 | 51.13 | 58.47 | 46.1 |
| | | 97–98 | 6987 | 0.78 | 66.4 | 41 | 36.58 |
| | | 98–99 | 3790 | 0.42 | 55.77 | | |
| | | 99–00 | 4797 | 0.53 | 59.52 | 38.35 | 38.21 |
| | MARIUS | 95–96 | 6933 | 0.77 | 36.15 | 42.2172 | 51.52 |
| | | 96–97 | 7322 | 0.82 | 51.13 | 58.47 | 46.1 |
| | | 97–98 | 6281 | 0.70 | 66.4 | 41 | 36.58 |
| | | 98–99 | 3458 | 0.39 | 55.77 | | |
| | | 99–00 | 4827 | 0.54 | 59.52 | 38.35 | 38.21 |
| | SOISSONS | 95–96 | 7295 | 0.81 | 36.15 | 42.2172 | 51.52 |
| | | 96–97 | 7832 | 0.87 | 51.13 | 58.47 | 46.1 |
| | | 97–98 | 6351 | 0.71 | 66.4 | 41 | 36.58 |
| | | 98–99 | 3482 | 0.39 | 55.77 | | |
| | | 99–00 | 4789 | 0.53 | 59.52 | 38.35 | 38.21 |
| | TREMIE | 95–96 | 8510 | 0.95 | 36.15 | | 51.52 |
| | | 96–97 | 8712 | 0.97 | 51.13 | 58.47 | 46.1 |
| | | 97–98 | 6870 | 0.77 | 66.4 | 41 | 36.58 |
| | | 98–99 | 3438 | 0.38 | 55.77 | | |
| | | 99–00 | 4703 | 0.52 | 59.52 | 38.35 | 38.21 |
| Vic | ETECHO | 95–96 | 8313 | 0.93 | 35.55 | | 50.64 |
| | | 96–97 | 6314 | 0.70 | 43.85 | 46.67 | 47.69 |
| | | 97–98 | 8133 | 0.91 | 58.07 | 64.45 | 49.19 |
| | | 98–99 | 5870 | 0.65 | 64.07 | 50.21 | 47.19 |
| | | 99–00 | 5834 | 0.65 | 74.2 | 49.99 | 48.81 |
| | MARIUS | 95–96 | 7370 | 0.82 | 35.55 | 42.2172 | 50.64 |
| | | 96–97 | 6262 | 0.70 | 43.85 | 46.67 | 47.69 |
| | | 97–98 | 7401 | 0.82 | 58.07 | 64.45 | 49.19 |
| | | 98–99 | 5000 | 0.56 | 64.07 | 50.21 | |
| | | 99–00 | 4616 | 0.51 | 74.2 | 49.99 | |
| | SOISSONS | 95–96 | 6807 | 0.76 | 35.55 | 42.2172 | 50.64 |
| | | 96–97 | 4894 | 0.55 | 43.85 | 46.67 | 47.69 |
| | | 97–98 | 8266 | 0.92 | 58.07 | 64.45 | 49.19 |
| | | 98–99 | 5571 | 0.62 | 64.07 | 50.21 | 47.19 |
| | | 99–00 | 5372 | 0.60 | 74.2 | 49.99 | 48.81 |
| | TREMIE | 95–96 | 8974 | 1.00 | 35.55 | | 50.64 |
| | | 96–97 | 7409 | 0.83 | 43.85 | 46.67 | 47.69 |
| | | 97–98 | 8210 | 0.91 | 58.07 | 64.45 | 49.19 |
| | | 98–99 | 4926 | 0.55 | 64.07 | 50.21 | 47.19 |
| | | 99–00 | 4401 | 0.49 | 74.2 | 49.99 | |

**TABLE 2**
**Yield, Relative Yield, and SWC (%FC) Data for Every Agroclimatic Station, Wheat Variety, and Year for the Agroclimatic Region Girona Interior**

| Station | Variety | Year | Yield (kg/ha) | Y | %FC (S–A) | %FC (S–H) | %FC (A–H) |
|---|---|---|---|---|---|---|---|
| Solsona | ETECHO | 95–96 | 7538 | 0.84 | 36.15 | 42.2172 | 51.52 |
| | | 96–97 | 7545 | 0.84 | 51.13 | 58.47 | 46.1 |
| | | 97–98 | 6987 | 0.78 | 66.4 | 41 | 36.58 |
| | | 98–99 | 3790 | 0.42 | 55.77 | | |
| | | 99–00 | 4797 | 0.53 | 59.52 | 38.35 | 38.21 |
| | MARIUS | 95–96 | 6933 | 0.77 | 36.15 | 42.2172 | 51.52 |
| | | 96–97 | 7322 | 0.82 | 51.13 | 58.47 | 46.1 |
| | | 97–98 | 6281 | 0.70 | 66.4 | 41 | 36.58 |
| | | 98–99 | 3458 | 0.39 | 55.77 | | |
| | | 99–00 | 4827 | 0.54 | 59.52 | 38.35 | 38.21 |
| | SOISSONS | 95–96 | 7295 | 0.81 | 36.15 | 42.2172 | 51.52 |
| | | 96–97 | 7832 | 0.87 | 51.13 | 58.47 | 46.1 |
| | | 97–98 | 6351 | 0.71 | 66.4 | 41 | 36.58 |
| | | 98–99 | 3482 | 0.39 | 55.77 | | |
| | | 99–00 | 4789 | 0.53 | 59.52 | 38.35 | 38.21 |
| | TREMIE | 95–96 | 8510 | 0.95 | 36.15 | | 51.52 |
| | | 96–97 | 8712 | 0.97 | 51.13 | 58.47 | 46.1 |
| | | 97–98 | 6870 | 0.77 | 66.4 | 41 | 36.58 |
| | | 98–99 | 3438 | 0.38 | 55.77 | | |
| | | 99–00 | 4703 | 0.52 | 59.52 | 38.35 | 38.21 |
| Vic | ETECHO | 95–96 | 8313 | 0.93 | 35.55 | | 50.64 |
| | | 96–97 | 6314 | 0.70 | 43.85 | 46.67 | 47.69 |
| | | 97–98 | 8133 | 0.91 | 58.07 | 64.45 | 49.19 |
| | | 98–99 | 5870 | 0.65 | 64.07 | 50.21 | 47.19 |
| | | 99–00 | 5834 | 0.65 | 74.2 | 49.99 | 48.81 |
| | MARIUS | 95–96 | 7370 | 0.82 | 35.55 | 42.2172 | 50.64 |
| | | 96–97 | 6262 | 0.70 | 43.85 | 46.67 | 47.69 |
| | | 97–98 | 7401 | 0.82 | 58.07 | 64.45 | 49.19 |
| | | 98–99 | 5000 | 0.56 | 64.07 | 50.21 | |
| | | 99–00 | 4616 | 0.51 | 74.2 | 49.99 | |
| | SOISSONS | 95–96 | 6807 | 0.76 | 35.55 | 42.2172 | 50.64 |
| | | 96–97 | 4894 | 0.55 | 43.85 | 46.67 | 47.69 |
| | | 97–98 | 8266 | 0.92 | 58.07 | 64.45 | 49.19 |
| | | 98–99 | 5571 | 0.62 | 64.07 | 50.21 | 47.19 |
| | | 99–00 | 5372 | 0.60 | 74.2 | 49.99 | 48.81 |
| | TREMIE | 95–96 | 8974 | 1.00 | 35.55 | | 50.64 |
| | | 96–97 | 7409 | 0.83 | 43.85 | 46.67 | 47.69 |
| | | 97–98 | 8210 | 0.91 | 58.07 | 64.45 | 49.19 |
| | | 98–99 | 4926 | 0.55 | 64.07 | 50.21 | 47.19 |
| | | 99–00 | 4401 | 0.49 | 74.2 | 49.99 | |

**TABLE 2 (continued)**

| Station | Variety | Year | Yield (kg/ha) | Y | %FC (S–A) | %FC (S–H) | %FC (A–H) |
|---|---|---|---|---|---|---|---|
| Ruidellots de la Selva | ETECHO | 94–95 | | | 35.11 | 25.5 | 36.75 |
| | | 95–96 | 6531 | 0.728 | 25.72 | 30.82 | 42.14 |
| | | 96–97 | 6306 | 0.703 | 57.21 | 58.71 | 48.15 |
| | | 97–98 | 7016 | 0.782 | 49.22 | 64.13 | 52.28 |
| | | 98–99 | 6087 | 0.678 | 44.31 | 55.56 | 48.89 |
| | | 99–00 | 5422 | 0.604 | 63.19 | 50.83 | 43.71 |
| | MARIUS | 94–95 | 5386 | 0.6 | 35.11 | 25.5 | 36.75 |
| | | 95–96 | 5667 | 0.631 | 25.72 | 30.82 | 42.14 |
| | | 96–97 | 6242 | 0.696 | 57.21 | 58.71 | 48.15 |
| | | 97–98 | 6668 | 0.743 | 49.22 | 64.13 | 52.28 |
| | | 98–99 | 6148 | 0.685 | 44.31 | 55.56 | 48.89 |
| | | 99–00 | 4406 | 0.491 | 63.19 | 50.83 | |
| | SOISSONS | 94–95 | 5740 | 0.64 | 35.11 | 25.5 | 36.75 |
| | | 95–96 | 4714 | 0.525 | 25.72 | 30.82 | 42.14 |
| | | 96–97 | 5452 | 0.608 | 57.21 | 58.71 | 48.15 |
| | | 97–98 | 7398 | 0.824 | 49.22 | 64.13 | 52.28 |
| | | 98–99 | 5626 | 0.627 | 44.31 | 55.56 | 48.89 |
| | | 99–00 | 4693 | 0.523 | 63.19 | 50.83 | 43.71 |
| | TREMIE | 94–95 | 6157 | 0.686 | 35.11 | 25.5 | 36.75 |
| | | 95–96 | 6335 | 0.706 | 25.72 | 30.82 | 42.14 |
| | | 96–97 | 6198 | 0.691 | 57.21 | 58.71 | 48.15 |
| | | 97–98 | 8495 | 0.947 | 49.22 | 64.13 | 52.28 |
| | | 98–99 | 5889 | 0.656 | 44.31 | 55.56 | 48.89 |
| | | 99–00 | 4896 | 0.546 | 63.19 | 50.83 | 43.71 |

**TABLE 3**
**Yield, Relative Yield, and SWC (%FC) Data for Every Agroclimatic Station, Wheat Variety, and Year for the Agroclimatic Region Secans Semifrescals**

| Station | Variety | Year | Yield (kg/ha) | Y | %FC (S–A) | %FC (S–H) | %FC (A–H) |
|---|---|---|---|---|---|---|---|
| Solsona | ETECHO | 95–96 | 7538 | 0.84 | 36.15 | 42.2172 | 51.52 |
| | | 96–97 | 7545 | 0.84 | 51.13 | 58.47 | 46.1 |
| | | 97–98 | 6987 | 0.78 | 66.4 | 41 | 36.58 |
| | | 98–99 | 3790 | 0.42 | 55.77 | | |
| | | 99–00 | 4797 | 0.53 | 59.52 | 38.35 | 38.21 |
| | MARIUS | 95–96 | 6933 | 0.77 | 36.15 | 42.2172 | 51.52 |
| | | 96–97 | 7322 | 0.82 | 51.13 | 58.47 | 46.1 |
| | | 97–98 | 6281 | 0.70 | 66.4 | 41 | 36.58 |
| | | 98–99 | 3458 | 0.39 | 55.77 | | |
| | | 99–00 | 4827 | 0.54 | 59.52 | 38.35 | 38.21 |
| | SOISSONS | 95–96 | 7295 | 0.81 | 36.15 | 42.2172 | 51.52 |
| | | 96–97 | 7832 | 0.87 | 51.13 | 58.47 | 46.1 |
| | | 97–98 | 6351 | 0.71 | 66.4 | 41 | 36.58 |
| | | 98–99 | 3482 | 0.39 | 55.77 | | |
| | | 99–00 | 4789 | 0.53 | 59.52 | 38.35 | 38.21 |
| | TREMIE | 95–96 | 8510 | 0.95 | 36.15 | | 51.52 |
| | | 96–97 | 8712 | 0.97 | 51.13 | 58.47 | 46.1 |
| | | 97–98 | 6870 | 0.77 | 66.4 | 41 | 36.58 |
| | | 98–99 | 3438 | 0.38 | 55.77 | | |
| | | 99–00 | 4703 | 0.52 | 59.52 | 38.35 | 38.21 |
| Vic | ETECHO | 95–96 | 8313 | 0.93 | 35.55 | | 50.64 |
| | | 96–97 | 6314 | 0.70 | 43.85 | 46.67 | 47.69 |
| | | 97–98 | 8133 | 0.91 | 58.07 | 64.45 | 49.19 |
| | | 98–99 | 5870 | 0.65 | 64.07 | 50.21 | 47.19 |
| | | 99–00 | 5834 | 0.65 | 74.2 | 49.99 | 48.81 |
| | MARIUS | 95–96 | 7370 | 0.82 | 35.55 | 42.2172 | 50.64 |
| | | 96–97 | 6262 | 0.70 | 43.85 | 46.67 | 47.69 |
| | | 97–98 | 7401 | 0.82 | 58.07 | 64.45 | 49.19 |
| | | 98–99 | 5000 | 0.56 | 64.07 | 50.21 | |
| | | 99–00 | 4616 | 0.51 | 74.2 | 49.99 | |
| | SOISSONS | 95–96 | 6807 | 0.76 | 35.55 | 42.2172 | 50.64 |
| | | 96–97 | 4894 | 0.55 | 43.85 | 46.67 | 47.69 |
| | | 97–98 | 8266 | 0.92 | 58.07 | 64.45 | 49.19 |
| | | 98–99 | 5571 | 0.62 | 64.07 | 50.21 | 47.19 |
| | | 99–00 | 5372 | 0.60 | 74.2 | 49.99 | 48.81 |
| | TREMIE | 95–96 | 8974 | 1.00 | 35.55 | | 50.64 |
| | | 96–97 | 7409 | 0.83 | 43.85 | 46.67 | 47.69 |
| | | 97–98 | 8210 | 0.91 | 58.07 | 64.45 | 49.19 |
| | | 98–99 | 4926 | 0.55 | 64.07 | 50.21 | 47.19 |
| | | 99–00 | 4401 | 0.49 | 74.2 | 49.99 | |

**TABLE 3 (continued)**

| Station | Variety | Year | Yield (kg/ha) | Y | %FC (S–A) | %FC (S–H) | %FC (A–H) |
|---|---|---|---|---|---|---|---|
| Ruidello | ETECHO | 94–95 | | | 35.11 | 25.5 | 36.75 |
| | | 95–96 | 6531 | 0.728 | 25.72 | 30.82 | 42.14 |
| | | 96–97 | 6306 | 0.703 | 57.21 | 58.71 | 48.15 |
| | | 97–98 | 7016 | 0.782 | 49.22 | 64.13 | 52.28 |
| | | 98–99 | 6087 | 0.678 | 44.31 | 55.56 | 48.89 |
| | | 99–00 | 5422 | 0.604 | 63.19 | 50.83 | 43.71 |
| | MARIUS | 94–95 | 5386 | 0.6 | 35.11 | 25.5 | 36.75 |
| | | 95–96 | 5667 | 0.631 | 25.72 | 30.82 | 42.14 |
| | | 96–97 | 6242 | 0.696 | 57.21 | 58.71 | 48.15 |
| | | 97–98 | 6668 | 0.743 | 49.22 | 64.13 | 52.28 |
| | | 98–99 | 6148 | 0.685 | 44.31 | 55.56 | 48.89 |
| | | 99–00 | 4406 | 0.491 | 63.19 | 50.83 | |
| | SOISSONS | 94–95 | 5740 | 0.64 | 35.11 | 25.5 | 36.75 |
| | | 95–96 | 4714 | 0.525 | 25.72 | 30.82 | 42.14 |
| | | 96–97 | 5452 | 0.608 | 57.21 | 58.71 | 48.15 |
| | | 97–98 | 7398 | 0.824 | 49.22 | 64.13 | 52.28 |
| | | 98–99 | 5626 | 0.627 | 44.31 | 55.56 | 48.89 |
| | | 99–00 | 4693 | 0.523 | 63.19 | 50.83 | 43.71 |
| | TREMIE | 94–95 | 6157 | 0.686 | 35.11 | 25.5 | 36.75 |
| | | 95–96 | 6335 | 0.706 | 25.72 | 30.82 | 42.14 |
| | | 96–97 | 6198 | 0.691 | 57.21 | 58.71 | 48.15 |
| | | 97–98 | 8495 | 0.947 | 49.22 | 64.13 | 52.28 |
| | | 98–99 | 5889 | 0.656 | 44.31 | 55.56 | 48.89 |
| | | 99–00 | 4896 | 0.546 | 63.19 | 50.83 | 43.71 |
| Calaf | ETECHO | 96–97 | 6200 | 0.991 | 66.22 | 74.77 | 49.43 |
| | | 97–98 | 3894 | 0.622 | 49.57 | 31.21 | 33.47 |
| | | 98–99 | 3528 | 0.564 | 52.92 | 34.13 | 36.87 |
| | | 99–00 | 3648 | 0.583 | 42.78 | 32.72 | 41.1 |
| | MARIUS | 96–97 | 5916 | 0.946 | 66.22 | 74.77 | |
| | | 97–98 | 3762 | 0.601 | 49.57 | 31.21 | 33.47 |
| | | 98–99 | 3278 | 0.524 | 52.92 | 34.13 | 36.87 |
| | | 99–00 | 3321 | 0.531 | 42.78 | 32.72 | 41.1 |
| | SOISSONS | 96–97 | 5846 | 0.934 | 66.22 | 74.77 | 49.43 |
| | | 97–98 | 3875 | 0.619 | 49.57 | 31.21 | 33.47 |
| | | 98–99 | 3040 | 0.486 | | 34.13 | 36.87 |
| | | 99–00 | 3530 | 0.564 | 42.78 | 32.72 | 41.1 |
| | TREMIE | 96–97 | 6105 | 0.976 | 66.22 | 74.77 | 49.43 |
| | | 97–98 | 3967 | 0.634 | 49.57 | 31.21 | 33.47 |
| | | 98–99 | 2722 | 0.435 | | 34.13 | 36.87 |
| | | 99–00 | 3368 | 0.538 | 42.78 | 32.72 | 41.1 |
| La Foradada | ETECHO | 96–97 | 4357 | 0.696 | 50.72 | 39.46 | 37.18 |
| | | 97–98 | 3343 | 0.534 | 49.86 | 30.13 | |
| | | 99–00 | 3578 | 0.572 | 38.32 | 26.89 | 34.66 |

**TABLE 3 (continued)**

| Station | Variety | Year | Yield (kg/ha) | Y | %FC (S–A) | %FC (S–H) | %FC (A–H) |
|---|---|---|---|---|---|---|---|
| | MARIUS | 95–96 | 4370 | 0.698 | 48.35 | | |
| | | 96–97 | 3780 | 0.604 | 50.72 | 39.46 | 37.18 |
| | | 97–98 | 3433 | 0.549 | 49.86 | 30.13 | 31.15 |
| | | 98–99 | 4018 | 0.642 | 52.39 | 32.77 | 39.37 |
| | | 99–00 | 3374 | 0.539 | 38.32 | 26.89 | 34.66 |
| | SOISSONS | 96–97 | 3841 | 0.614 | 50.72 | 39.46 | 37.18 |
| | | 97–98 | 3406 | 0.544 | 49.86 | 30.13 | 31.15 |
| | | 98–99 | 4677 | 0.747 | 52.39 | 32.77 | 39.37 |
| | | 99–00 | 3780 | 0.604 | 38.32 | 26.89 | 34.66 |
| | TREMIE | 96–97 | 4483 | 0.716 | 50.72 | 39.46 | 37.18 |
| | | 97–98 | 3066 | 0.49 | 49.86 | 30.13 | 31.15 |
| | | 98–99 | 5623 | 0.899 | | | 39.37 |
| | | 99–00 | 3532 | 0.564 | 38.32 | 26.89 | 34.66 |
| Santa Coloma | MARIUS | 94–95 | 2827 | 0.452 | 39.52 | 32.23 | |
| | SOISSONS | 94–95 | 3153 | 0.504 | 39.52 | 32.23 | 42.92 |
| | TREMIE | 94–95 | 3482 | 0.556 | 39.52 | 32.23 | 42.92 |

**TABLE 4**
**Mean Temperature for Every Agroclimatic Region, Classified by Station, Year, and Period**

| Agroclimatic Region | Station | Year | Mean T(ºC) (S–A) | Mean T(ºC) (S–H) | Mean T(ºC) (A–H) |
|---|---|---|---|---|---|
| Secans Semifrescals | Calaf | 94–95 | 10 | 11 | 18 |
| | | 95–96 | 10 | 11 | 18 |
| | | 96–97 | 10 | 11 | 18 |
| | | 97–98 | 10 | 11 | 19 |
| | | 98–99 | 9 | 10 | 19 |
| | | 99–00 | 9 | 10 | 19 |
| | La Foradada | 94–95 | 10 | 11 | 19 |
| | | 95–96 | 10 | 12 | 19 |
| | | 96–97 | 11 | 12 | 19 |
| | | 97–98 | 10 | 12 | 20 |
| | | 98–99 | 9 | 10 | 20 |
| | | 99–00 | 9 | 10 | 21 |
| Secans Frescals | Vic | 95–96 | 7 | 9 | 15 |
| | | 96–97 | 9 | 10 | 16 |
| | | 97–98 | 8 | 9 | 16 |
| | | 98–99 | 7 | 9 | 17 |
| | | 99–00 | 7 | 9 | 17 |
| | Solsona | 95–96 | 8 | 9 | 16 |
| | | 96–97 | 9 | 10 | 16 |
| | | 97–98 | 8 | 9 | 17 |
| | | 98–99 | 7 | 9 | 18 |
| | | 99–00 | 7 | 9 | 17 |
| Girona Interior | Ruidellots de la Selva | 94–95 | 10 | 11 | 17 |
| | | 95–96 | 10 | 11 | 17 |
| | | 96–97 | 11 | 12 | 18 |
| | | 97–98 | 10 | 11 | 18 |
| | | 98–99 | 10 | 11 | 18 |
| | | 99–00 | 9 | 11 | 18 |

**TABLE 5**
**Accumulated Global Radiation for Every Agroclimatic Region, Classified by Station, Year, and Period**

| Agroclimatic Region | Station | Year | Global Radiation (MJ $m^{-2}$) (S–A) | Global Radiation (MJ $m^{-2}$) (S–H) | Global Radiation (MJ $m^{-2}$) (A–H) |
|---|---|---|---|---|---|
| Secans Semifrescals | Calaf | 94–95 | 2160 | 2755 | 1225 |
| | | 95–96 | 2004 | 2630 | 1226 |
| | | 96–97 | 1990 | 2535 | 1089 |
| | | 97–98 | 2319 | 3087 | 1366 |
| | | 98–99 | 2433 | 3163 | 1377 |
| | | 99–00 | 2404 | 3124 | 1386 |
| | La Foradada | 94–95 | 2604 | 3299 | 1418 |
| | | 95–96 | 2610 | 3382 | 1514 |
| | | 96–97 | 2579 | 3269 | 1372 |
| | | 97–98 | 2589 | 3350 | 1433 |
| | | 98–99 | 2560 | 3317 | 1418 |
| | | 99–00 | 2649 | 3421 | 1494 |
| Secans Frescals | Vic | 95–96 | 2451 | 3170 | 1445 |
| | | 96–97 | 2658 | 3317 | 1362 |
| | | 97–98 | 2565 | 3310 | 1386 |
| | | 98–99 | 2732 | 3447 | 1414 |
| | | 99–00 | 2688 | 3425 | 1386 |
| | Solsona | 95–96 | 2383 | 3114 | 1439 |
| | | 96–97 | 2510 | 3174 | 1342 |
| | | 97–98 | 2411 | 3172 | 1375 |
| | | 98–99 | 2522 | 3268 | 1404 |
| | | 99–00 | 2546 | 3299 | 1428 |
| Girona Interior | Ruidellots de la Selva | 94–95 | 2039 | 2590 | 1173 |
| | | 95–96 | 2264 | 2821 | 1229 |
| | | 96–97 | 2408 | 2956 | 1180 |
| | | 97–98 | 2370 | 3028 | 1260 |
| | | 98–99 | 2529 | 3166 | 1265 |
| | | 99–00 | 2437 | 3072 | 1204 |

**TABLE 6**
**Accumulated Precipitations (ΣN) for Every Agroclimatic Region, Classified by Station, Year, and Period**

| Agroclimatic Region | Station | Year | ΣN (mm) (S–A) | ΣN (mm) (S–H) | ΣN (mm) (A–H) |
|---|---|---|---|---|---|
| Secans Semifrescals | Calaf | 94–95 | 114.1 | 168.3 | 86.9 |
| | | 95–96 | 345.8 | 451.6 | 168.7 |
| | | 96–97 | 328.3 | 431.6 | 117.4 |
| | | 97–98 | 211.3 | 223.2 | 37.4 |
| | | 98–99 | 252 | 256.7 | 79.5 |
| | | 99–00 | 169.8 | 236.8 | 123 |
| | La Foradada | 94–95 | 144.1 | 155.6 | 27.9 |
| | | 95–96 | 262.1 | 278.6 | 26.1 |
| | | 96–97 | 301.5 | 367.7 | 82 |
| | | 97–98 | 266.8 | 269.4 | 38.3 |
| | | 98–99 | 273.1 | 279.9 | 128 |
| | | 99–00 | 173.7 | 235.3 | 101.3 |
| Secans Frescals | Vic | 95–96 | 527.4 | 605.6 | 154 |
| | | 96–97 | 518 | 626 | 127.4 |
| | | 97–98 | 366.6 | 437 | 148.6 |
| | | 98–99 | 299.8 | 371.2 | 148.8 |
| | | 99–00 | 315.2 | 366.2 | 155.8 |
| | Solsona | 95–96 | 521.2 | 612.8 | 167.8 |
| | | 96–97 | 442 | 533 | 116.4 |
| | | 97–98 | 265.6 | 272 | 41.8 |
| | | 98–99 | 241.2 | 257.4 | 94.6 |
| | | 99–00 | 264.4 | 266.8 | 76.8 |
| Girona Interior | Ruidellots de la Selva | 94–95 | 48 | 48 | 0.2 |
| | | 95–96 | 678.2 | 739.6 | 69 |
| | | 96–97 | 425 | 536.6 | 118.2 |
| | | 97–98 | 451.1 | 514.1 | 167 |
| | | 98–99 | 508.2 | 567.6 | 148.2 |
| | | 99–00 | 301.6 | 377.6 | 96 |

**TABLE 7**
**Accumulated Potential Evapotranspiration (ΣETP) for Every Agroclimatic Region, Classified by Station, Year, and Period**

| Agroclimatic Region | Station | Year | ΣETP (mm) (S–A) | ΣETP (mm) (S–H) | ΣETP (mm) (A–H) |
|---|---|---|---|---|---|
| Secans Semifrescals | Calaf | 94–95 | 315 | 448 | 255 |
| | | 95–96 | 282 | 427 | 255 |
| | | 96–97 | 305 | 428 | 232 |
| | | 97–98 | 325 | 515 | 303 |
| | | 98–99 | 342 | 514 | 307 |
| | | 99–00 | 339 | 511 | 308 |
| | La Foradada | 94–95 | 388 | 553 | 313 |
| | | 95–96 | 385 | 574 | 336 |
| | | 96–97 | 407 | 569 | 307 |
| | | 97–98 | 378 | 574 | 331 |
| | | 98–99 | 367 | 553 | 330 |
| | | 99–00 | 387 | 581 | 353 |
| Secans Frescals | Vic | 95–96 | 285 | 431 | 260 |
| | | 96–97 | 358 | 490 | 255 |
| | | 97–98 | 308 | 467 | 265 |
| | | 98–99 | 331 | 480 | 281 |
| | | 99–00 | 319 | 477 | 275 |
| | Solsona | 95–96 | 286 | 438 | 267 |
| | | 96–97 | 340 | 476 | 257 |
| | | 97–98 | 288 | 458 | 273 |
| | | 98–99 | 313 | 478 | 291 |
| | | 99–00 | 316 | 479 | 291 |
| Girona Interior | Ruidellots de la Selva | 94–95 | 295 | 412 | 229 |
| | | 95–96 | 317 | 443 | 245 |
| | | 96–97 | 361 | 481 | 242 |
| | | 97–98 | 335 | 483 | 261 |
| | | 98–99 | 352 | 494 | 267 |
| | | 99–00 | 336 | 482 | 257 |
